# Bioassay-Guided Isolation of Antiplasmodial Compounds from *Hypericum lanceolatum* Lam. (Hypericaceae) and Their Cytotoxicity and Molecular Docking

**DOI:** 10.1155/2023/4693765

**Published:** 2023-05-29

**Authors:** Gervais Mouthé Happi, Sikiru Akinyeye Ahmed, Guy Paulin Mouthé Kemayou, Shina Salau, Liliane Clotilde Dzouemo, Klev Gaïtan Sikam, Mireille Towa Yimtchui, Jean Duplex Wansi

**Affiliations:** ^1^Department of Chemistry, Higher Teacher Training College, The University of Bamenda, P.O. Box 39, Bambili, Cameroon; ^2^Department of Chemistry and Industrial Chemistry, Kwara State University, Malete, P.M.B 1530, Ilorin 23431, Nigeria; ^3^Department of Organic Chemistry, Faculty of Sciences, University of Yaounde I, P.O. Box 812, Yaounde, Cameroon; ^4^Department of Chemistry, Faculty of Sciences, University of Douala, P.O. Box 24157, Douala, Cameroon

## Abstract

In Cameroon, malaria is still the cause of several deaths yearly and leading to the continued search for new potent leads to fight against *Plasmodium falciparum*. Medicinal plants like *Hypericum lanceolatum* Lam. are introduced in local preparations for the treatment of affected people. The bioassay-guided fractionation of the crude extract of the twigs and stem bark of *H. lanceolatum* Lam. led to the identification of the dichloromethane-soluble fraction as the most active (with 32.6% of the parasite *P. falciparum* 3D7 survival) which was further purified by successive column chromatography to obtain four compounds identified by their spectrometric data as two xanthones 1,6-dihydroxyxanthone (**1**) and norathyriol (**2**) and two triterpenes betulinic acid (**3**) and ursolic acid (**4**). In the antiplasmodial assay against *P. falciparum* 3D7, the triterpenoids **3** and **4** displayed the most significant potencies with IC_50_ values of 2.8 ± 0.8 *μ*g/mL and 11.8 ± 3.2 *μ*g/mL, respectively. Furthermore, both compounds were also the most cytotoxic against P388 cell lines with IC_50_ values of 6.8 ± 2.2 *μ*g/mL and 2.5 ± 0.6 *μ*g/mL, respectively. Further insights on the inhibition method of the bioactive compounds and their drug-likeness were obtained from their molecular docking and ADMET studies. The results obtained help in identifying additional antiplasmodial agents from *H. lanceolatum* and support its use in folk medicine for the treatment of malaria. The plant might be considered as a promising source of new antiplasmodial candidates in new drug discovery.

## 1. Introduction

For several centuries, malaria remains a significant threat to public health around the world and especially in developing countries which are still struggling to control and eradicate it [1]. *Plasmodium falciparum* is one of the parasites that cause the most severe forms of disease around the world [2]. The observed resistance of *P. falciparum* to prescribed antimalarial drugs is the main challenge that causes the increased incidence of malaria around the globe [3, 4]. However, the literature survey indicated that several compounds with antimalarial potential have been isolated from natural sources including plants, mushrooms, or microbes [2, 5, 6]. In malaria-endemic countries like Cameroon, the local population refers to medicinal plants for their treatment, and an important number of plants from Cameroonian flora have been reported as promising sources of antiplasmodial compounds [7–12].

Hypericaceae is a large family of plants comprising eleven genera and almost 584 accepted species [13]. The genera *Hypericum*, *Harungana*, *Vismia*, and *Psorospermum* are amongst the most encountered and widely used by the traditional healers and local population for the management of numerous affections including malaria, typhoid fever, wounds, inflammatory diseases, or microbial infections [14–16]. A recent paper has reported the *Hypericum* genus as a significant source of natural bioactive compounds with a large range of traditional use for the treatment of illnesses like fever, jaundice, pain, lung abscesses, poisoning from venomous animal bites, hepatic disorders, and dysentery [16]. Likewise, the plant *Harungana madagascariensis*, the sole species of the genus *Harungana*, is commonly used for the treatment of malaria in Cameroonian folk medicine, and its use is further supported by the antiprotozoal activities of its extracts reported in literature [17, 18]. In their recent review, Tepa et al. have compiled an updated list of Cameroonian medicinal plants introduced in local preparations of medicines for malaria-affected persons as well as their in vitro potency against *P. falciparum* strains [19]. From their meta-analysis, they concluded that the plant *Dacryodes edulis* (African pear) (Burseraceae) represents a significant opportunity to be considered in the treatment of uncomplicated malaria affection; furthermore, plants like *Entandrophragma congoënse* (Meliaceae), *Xylopia africana* (Annonaceae), *Vernonia guineensis* (Asteraceae), or *Strychnos icaja* (Loganiaceae) displayed interesting active compounds with promising activities in the fight against *P. falciparum* [19].

*Hypericum lanceolatum* Lam. (Hypericaceae) is a rare species found in the mountainous region of West Cameroon [20]. The plant is widely used in traditional medicine by the local population for the treatment of malaria and several other ailments [21, 22]. Briefly, it is reported that the solution obtained from the maceration of its leaves with palm wine can help for the treatment of skin infections and epilepsies while its roots can be boiled in water and taken for the treatment of microbial infections like dysentery, venereal diseases, and gastrointestinal disorders [21, 22].

Considering the few number of reports on phytochemical and biological investigations of the plant which is very rare and is important in traditional medicine, we have carried out further investigations of the twigs and stem bark of *H. lanceolatum* as continuity of our ongoing project on the identification of antiplasmodial lead compounds from Cameroonian medicinal plants [7–9]. The methanolic crude extract was partitioned using four organic solvents and tested against *P. falciparum* 3D7. The results showed that the dichloromethane-soluble fraction showed the lowest rate of parasite survival (most active) and was further investigated. We report herein the isolation and characterization of the bioactive compounds from the active fraction as well as their cytotoxicity, molecular docking, and ADMET analyses.

## 2. Materials and Methods

### 2.1. Plant Material

The twigs and stem bark of *H. lanceolatum* Lam. were harvested in February 2021 at Balatachi (5° 37′ 42^″^ N, 10° 12′ 03^″^ E, 1580 m), Bamboutos Subdivision, West Region, Cameroon. Its identification was done by a botanist at the National Herbarium of Cameroon by morphological comparison of the collected plant material with the previous one available in the database of the herbarium under voucher number 32356/HNC.

### 2.2. Extraction and Bioassay-Guided Fractionation

**Figure 1:**
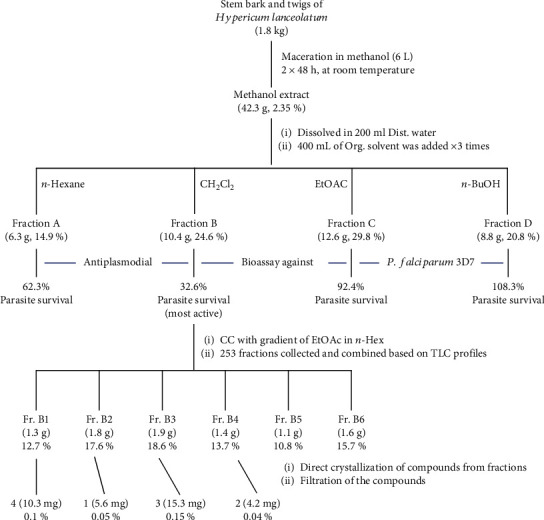
Flowchart of the isolation of bioactive compounds from *H. lanceolatum.*

The extraction and isolation of the bioactive compounds were carried out following the chart in Figure 1. Briefly, the collected stem bark and twigs of *H. lanceolatum* were cut, air-dried, and ground to afford almost 1.8 kg of powder which were macerated twice in 6 L of methanol at room temperature for 48 h, each. After filtration and removal of the solvent under reduced pressure, we obtain 42.3 g of crude extract which was dissolved in 200 mL of distilled water and partitioned with *n*-hexane, dichloromethane, ethyl acetate, and *n*-butanol (three times each using 400 mL of organic solvent) to afford four main fractions A (6.3 g), B (10.4 g), C (12.6), and D (8.8 g), respectively. The four major fractions A–D were subjected to antiplasmodial assay, in which fraction B (dichloromethane-soluble fraction) was found to be the most active with 32.6% of parasite survival.

Further purification of fraction B (10.2 g) with silica gel column chromatography eluting with a gradient of ethyl acetate in *n*-hexane from the proportion 4 : 1 to 2 : 3 led to the collection of 253 fractions of 100 mL each, which were combined into six subfractions B1–B6. Four pure compounds (**1**–**4**) were filtered after their precipitation in four subfractions. For instance, ursolic acid (**4**, 10.3 mg) was obtained as a white powder in B1 (1.3 g, *n*-hexane/ethyl acetate 3 : 1), while an orange solid 1,6-dihydroxyxanthone (**1**, 5.6 mg) was precipitated in B2 (1.8 g, *n*-hexane/ethyl acetate 7 : 3). Likewise, another white powder identified as betulinic acid (**3**, 15.3 mg) was formed in B3 (1.9 g, *n*-hexane/ethyl acetate 13 : 7), and norathyriol (**2**, 4.2 mg) was a brown deposit in B4 (1.4 g, *n*-hexane/ethyl acetate 11 : 9).

### 2.3. Identification of Isolated Compounds

The four isolated compounds have been identified using their NMR data. Briefly, their ^1^H and ^13^C-NMR spectra were recorded on a Bruker Advance III 500 MHz NMR spectrometer (Bruker, Rheinstetten, Germany) equipped with a 5 mm cryogenic DCH (^1^H/^13^C) probe. Chemical shifts are reported in parts per million (*δ*) using tetramethylsilane (TMS) (Sigma-Aldrich, Munich, Germany) as the internal standard, while coupling constants (*J*) were measured in hertz. Column chromatography was carried out on silica gel 230-400 mesh (Merck, Darmstadt, Germany) and silica gel 70-230 mesh (Merck).

### 2.4. Antiplasmodial Assay

The antiplasmodial evaluation of the fractions and pure compounds has been done following the protocol described in our recent publication [7].

#### 2.4.1. Culture of the Parasite

*Plasmodium falciparum* 3D7 strain (chloroquine-sensitive) was obtained from the Biodefense and Emerging Infections (BEI) Research Resources (Manassas, VA) and were cultured in fresh O^+^ human red blood cells at 3% (*v*/*v*) hematocrit in RPMI 1640 culture media containing glutamax and NaHCO_3_ (Gibco, UK), supplemented with 25 mM HEPES (Gibco, UK), 1x hypoxanthine (Gibco, USA), 20 *μ*g/mL gentamicin (Gibco, China), and 0.5% Albumax II (Gibco, USA). The obtained parasitic cultures were treated with 5% D-sorbitol to obtain only ring-stage parasitized cells, and 1% ring-stage of parasitemia was maintained for antimalarial assay.

#### 2.4.2. *In Vitro* Antiplasmodial Assay

**Figure 2:**
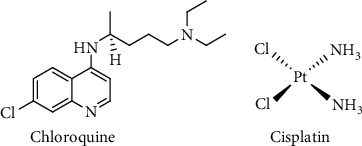
Structures of reference drugs used in this study.

The fractions obtained from the partition of the crude extracts as well as the compounds isolated from the most active fraction were all tested *in vitro* for their antiplasmodial activities using the schizont maturation inhibition assay method. Briefly, the samples to be tested were dissolved into dimethyl sulfoxide (DMSO) to obtain the stock solutions (10 mM) which were subsequently diluted in triplicate with an RPMI medium which have been introduced in the prepared tested samples and parasite cultures (1% parasitemia and 1.5% hematocrit). After the successive dilution process and the mixture of samples with parasite cultures, the final concentration of DMSO per 100 *μ*L culture per well was 0.1% while chloroquine (Figure 2) was used as the reference drug at a range of 1–0.0078 *μ*M each and dimethyl sulfoxide (0.1%) was used as the negative control. All the prepared solutions were incubated for 72 h at 37°C and 5% CO_2_-humidified atmosphere. Upon incubation, plates were checked under a microscope for the presence of trophozoite stage through evaluation of Giemsa-stained smears. The schizont inhibition (%) of each concentration of prepared samples was evaluated to determine the level of parasitemia using the formulae: schizont inhibition (%) = no.of schizonts (control)–no.of schizonts (test)/no.of schizonts (control) × 100. Subsequently, the concentration that inhibits 50% of parasite growth (IC_50_) was determined and given as mean ± SD (*n* = 3).

### 2.5. In Vitro Cytotoxicity Assay on P388 Cell Line

The *in vitro* study of the cytotoxicity potency of the isolated compounds was performed using the P388 (mouse leukaemia) cell line which was obtained from the American Type Culture Collection (ATCC) (Rockville, MD, USA). The cells were arranged in 96-well plates at a density of 10^4^ cells per well containing 100 mL of culture medium and then cultured for 24 h in the opti-MEM medium supplemented with 2 mM glutamine (Gibco, Grand Island, NY, USA), streptomycin (50 *μ*g/mL), penicillin (50 U/mL), and 5% fetal calf serum (Gibco). After the cell cultures were maintained at 37°C in an atmosphere saturated with 5% CO_2_, the four tested compounds prepared at the concentration of 1 mg/mL by dissolving in the mixture DMSO/culture medium (1 : 9) were diluted in cell culture to reach the required concentrations (ranging from 1 to 100 *μ*g/mL). The *in vitro* cytotoxic activity of the tested compounds was evaluated using the MTT assay for leukaemia cells. Cisplatin (Figure 2) was used as the positive control, the experiment was done in triplicate, and the results (IC_50_) were expressed as mean ± SD (*n* = 3).

### 2.6. Statistical Analysis

The data from biological activities were treated using one-way ANOVA and SigmaPlot 12.5 software. The level of significance in all statistical tests was *p* < 0.05.

### 2.7. Selectivity Index (SI)

The selectivity index (SI) was calculated to access the selectivity of the compounds to *P. falciparum* and is expressed as the ratio between the cytotoxic and antiparasitic activity (IC_50_) of each tested sample.

### 2.8. Computational Methodology

#### 2.8.1. Ligand Preparations

Four compounds that were isolated from *H. lanceolatum* were drawn with the ChemDraw® Ultra software [23]⁠ and optimized using the Avogadro® software [24]⁠ at the Molecular Mechanics Force Field ‘99 (MMFF99) level. Then, ligands were recorded in *.pdb* format with a specified hydrogen state. Chloroquine® was the standard used for this study.

#### 2.8.2. Receptor Preparations

The selected target for the *in silico* investigation is the malaria *P. falciparum* receptor (5TBO.pdb).

The receptor was retrieved from the RCSB database at http://www.rcsb.org.

Using the BIOVIA® DS version 2021 software [25]⁠⁠, the receptor was processed by removing water molecules and adding polar hydrogens as well as polar charges. Following that, the files were stored in a *.pdbqt* format.

#### 2.8.3. Docking Protocol

The *.pdb* binding site for the receptor was consulted from the Computed Atlas of Surface Topography of Proteins (CASTp) webserver [26]⁠. The binding sites revealed a catalytic triad of residues, A:249, A:342, and A:429.

#### 2.8.4. Molecular Docking

Vina/PyRx® software tools were used to simulate molecular docking [27, 28]⁠. We measured the receptor-binding affinities of isolated metabolites from *H. lanceolatum*.

#### 2.8.5. Graphical Illustrations

The ligand interaction graphics from the Vina score were taken from the BIOVIA® DS version 2021 software [25]⁠⁠, on an 8-core Debian 10 machine while the R statistical programming language's ggplot2 package [29]⁠ was used to create all graphical plots.

#### 2.8.6. ADMET Property Prediction

The SMILES index formula of each isolated molecule in the ADMETLab 2.0 [30]⁠ web server allowed for the prediction of the absorption, distribution, metabolism, excretion, and toxicity properties.

## 3. Results and Discussion

### 3.1. Phytochemical Study

**Figure 3:**
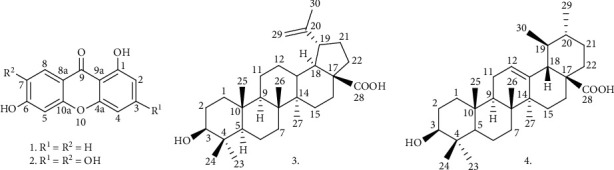
Chemical structures of compounds (**1**–**4**) isolated from *H. lanceolatum.*

The antiplasmodial bioassay-guided investigation of *H. lanceolatum* twigs and stem bark methanolic extract led to the identification of the dichloromethane-soluble fraction as the most active one from which four distinct compounds (Figure 3) have been isolated as major leads with significant yields in the range of 0.05–0.15% of the dichloromethane active subextract (Table 1). The structure elucidation of the isolated compounds was conducted by exploration of their spectral data (see Figures S1–S8 on supplementary data) which allow their characterization as two xanthones including 1,6-dihydroxyxanthone (**1**) [31] and norathyriol (**2**) [32] as well as two triterpenes, namely, betulinic acid (**3**) [14, 33] and ursolic acid (**4**) [34].

Xanthones represent one of the main classes of compounds reported from the family Hypericaceae [15]. Unsurprisingly, two xanthones (**1** and **2**) have been obtained from the most active fraction. Although both compounds **1** and **2** are reported for the first time from the genus *Hypericum*, ten xanthones and derivatives have been reported from the previous phytochemical investigations of *H. lanceolatum* [21].

### 3.2. Antiplasmodial Activity

The major compound betulinic acid (**3**) obtained with the highest yield of 0.15% (Table 1) was also found to be the most active compound in the fraction with an IC_50_ value of 2.8 ± 0.8 *μ*g/mL against *P. falciparum* 3D7 and SI of 2.4 (Table 2). The compound has been previously described in *H. lanceolatum* [21] and also reported as one of its antimalarial agents with potency evaluated against the strains W2mef and SHF4 of *P. falciparum* [22].

The second triterpene ursolic acid (**4**) was obtained from the active fraction with a yield of 0.1% and a potency in terms of IC_50_ equal to 11.8 ± 3.2 *μ*g/mL and the lowest SI of 0.2. Our recent report on the investigations of Cameroonian medicinal plants for their antiplasmodial metabolites indicated that ursolic acid (**4**) possessed a weak antiplasmodial potency and inhibited the *Plasmodium* parasite through interactions with *Plasmodium* dihydroorotate dehydrogenase indicating important residue-hydrogen bond interactions with ASN274 (3.07), CYS276 (3.00), and GLY277 (2.32) [7].

Compounds **1** and **2** were obtained with the lowest yields and were also the less active compounds from the fraction. That observation indicated that the potency of the fraction is supported by the activity of its major compounds identified as the two triterpenes here. Therefore, a synergistic action might be associated with their action mechanism in inhibiting the parasite *P. falciparum* 3D7.

### 3.3. Cytotoxicity Assay

**Figure 4:**
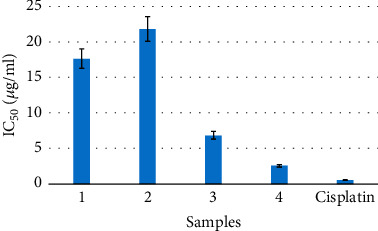
Cytotoxicity of tested compounds (**1**–**4**) and cisplatin against P388 cell lines.

The cytotoxicity test using the MTT assay method against P388 murine leukaemia cells (Table 2 and Figure 4) showed strong cytotoxic activity with IC_50_ values ranging from 2.5 *μ*g/mL for compound **4** to 21.8 *μ*g/mL for compound **2**. It is important to indicate that the two most active compounds **3** and **4** displayed the highest cytotoxicity and the SI values of 2.4 and 0.2, respectively, which obviously represented the highest and lowest SI values of this study.

However, even if the antiplasmodial test showed some promising results in searching for new antiplasmodial agents, it is important to notice that the high cytotoxicity of the active compounds against this tumour cell line might limit their use as a potential treatment. This observation can be linked to the low SI values of tested compounds (<10) which indicate an unfavourable safety window between the effective concentration against the parasite and the toxic concentration to the cell line [35]. Therefore, further investigations are required to increase the potency of the potent candidates isolated within this study and also to reduce their toxicity for the safe development of new potent antiplasmodial drugs.

### 3.4. Molecular Docking

Further insights on the antiplasmodial potency of the isolated compounds and evaluation of their drug-likeness have been provided by their *in silico* inhibition of the *P. falciparum* dihydroorotate dehydrogenase (PDB code: 5TBO) which is an important target enzyme associated to *P. falciparum* 3D7 used in this study for *in vitro* tests of isolated compounds [36].

**Figure 5:**
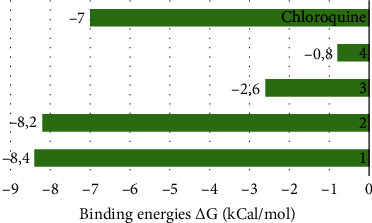
Binding affinity score of *H. lanceolatum* extracts against 5TBO receptor.

**Figure 6:**
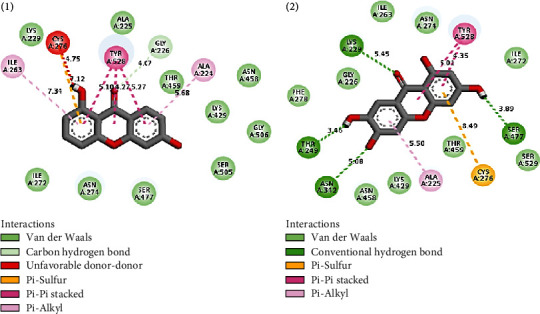
Interactions of compounds **1** and **2** with 5TBO receptor in its binding site.

**Figure 7:**
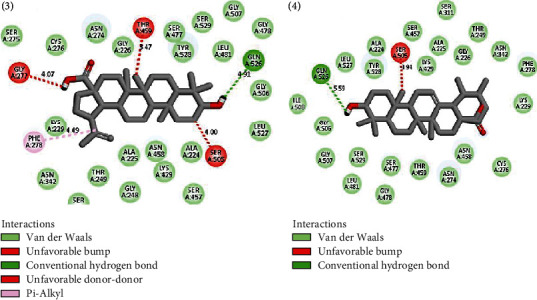
Interactions of compounds **3** and **4** with 5TBO receptor in its binding site.

**Figure 8:**
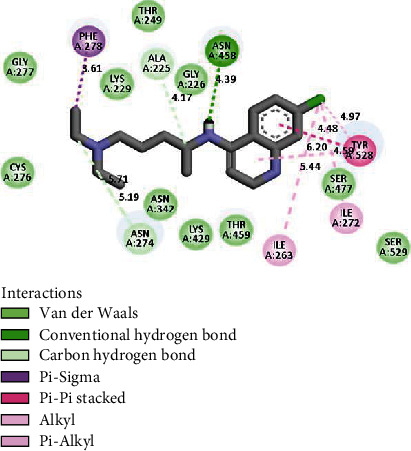
Interactions of chloroquine with 5TBO receptor in its binding site.

Table 3 and Figure 5 describe the binding actions of the analyzed compounds with the binding energy associated with them. The result infers that the xanthones **1** and **2** (-8.4 kcal/mol and -8.2 kcal/mol, respectively) performed better than the standard Chloroquine® with the binding energy of -7.0 kcal/mol, but their *in vitro* potencies were lower than those of triterpenoids **3** and **4** (Table 2). This might be explained by the nature of the interactions of the compounds in the binding site of the receptor which is mostly *π*–*π* stacked bonds for the xanthones and conventional hydrogen bonds for the triterpenoids. Figures 6–8 better illustrate the standing of the isolated bioactive compounds performing better in binding activity than Chloroquine®, the standard drug used in this study.

From those results, we can suggest that the interactions of ligands with GLN526 (4.91 and 5.59), THR249 (3.46), SER477 (3.89), and ASN342 (5.08) through conventional hydrogen bonds in the binding site of *P. falciparum* dihydroorotate dehydrogenase might play an important role in the inhibitory mechanism of *P. falciparum* 3D7 (Figures 6 and 7).

### 3.5. ADMET Studies

The drug-likeness of the bioactive compounds isolated from *H. lanceolatum* was evaluated, and the results are compiled in Table 4 describing their adsorption, distribution, metabolism, excretion, and toxicity (ADMET) properties. The results show that the xanthones **1** and **2** have good absorptions properties with excellent water solubilities as advised by Falade et al. [37]⁠ that a potential drug should have water solubility within the range of 7 to 3 g/dm^3^. Likewise, compounds **1**–**4** are reported to possess a noncompetitive drug metabolic mechanism as they are seen to not inhibit any of the cytochrome P450 enzymes. Based on their computational validation, compounds **1**–**4** are very less toxic if used as an antimalarial drug as the standard drug Chloroquine® (Table 4).

These results as for the *in vitro* cytotoxicity studies showed that the compounds deserve further investigations on further (tumour and normal) cell lines before a further conclusion on their safe use.

## 4. Conclusion

This paper deals with the antiplasmodial bioassay-guided investigations of the twigs and stem bark of *H. lanceolatum* from which four major active compounds identified as 1,6-dihydroxyxanthone (**1**), norathyriol (**2**), betulinic acid (**3**), and ursolic acid (**4**) have been isolated and displaying good to moderate antiplasmodial activity against the chloroquine-sensitive strain *P. falciparum* 3D7 as well as strong cytotoxicity against the P388 cell line. Furthermore, the *in silico* antiplasmodial evaluation of the isolated compounds was performed to provide further evidence of their action mechanism that could support their potency, while the ADMET study of the isolated compounds showed that they displayed favourable drug-likeness parameters as leads. In addition to complementing the previous results reported, the results justify the use of the plant in folk medicine for the treatment of malaria and related symptoms indicating that the plant *H. lanceolatum* is a promising source of candidates requiring further pharmacological and pharmacokinetic investigations in the development of new potent drugs or phytomedicines for malaria.

## Figures and Tables

**Table 1:** Yield of isolated bioactive compounds in the active fraction.

Compounds	Yield
1	0.05%
2	0.04%
3	0.15%
4	0.10%

**Table 2:** Antiplasmodial and cytotoxicity activities of extracts and isolated compounds.

Compounds	Antiplasmodial (*Pf* 3D7)		Cytotoxicity (P388)	SI
	Parasite survival	IC_50_ (*μ*g/mL)	IC_50_ (*μ*g/mL)	
F1	62.3%	—	—	—
F2	32.6%	—	—	—
F3	92.4%	—	—	—
F4	108.3%	—	—	—
1	—	33.6 ± 0.1	17.6 ± 0.7	0.5
2	—	52.2 ± 4.6	21.8 ± 0.3	0.4
3	—	2.8 ± 0.8	6.8 ± 2.2	2.4
4	—	11.8 ± 3.2	2.5 ± 0.6	0.2
Chloroquine	—	0.07 ± 0.01	—	—
Cisplatin	—	—	0.5 ± 0.2	—

% parasite survival at 10 *μ*g/mL. F1: hexane-soluble fraction; F2: CH_2_Cl_2_-soluble fraction; F3: EtOAC-soluble fraction; F4: *n*-butanol-soluble fraction; SI: selectivity index.

**Table 3:** Binding energy to the antiplasmodium (5TBO) receptor.

Compounds	Binding energy (Δ*G*) kcal/mol
1	-8.4
2	-8.2
3	-2.6
4	-0.8
Chloroquine	-7.0

**Table 4:** ADMET prediction results of isolated compounds of *H. lanceolatum* extract.

Absorption and distribution	1	2	3	4	Chloroquine
BBB (±)	++	+	+	—	+
GIA (±)	Good	Very good	Very poor	Very good	Good
Log S	–6.44	–4.9	–7.06	–4.94	-1.65
CYP450 2C19 inhibitor	No	No	No	No	No
CYP450 1A2 inhibitor	No	No	No	No	No
CYP450 3A4 inhibitor	No	No	No	No	No
CYP450 2C9 inhibitor	No	No	No	No	No
CYP450 2D6 inhibitor	No	No	No	No	No
Toxicity					
AMES mutagenesis	No	No	No	No	No
Acute oral/toxicity rating	Low	Low	Low	Low	Low
hERG toxicity	None	None	None	None	None
Carcinogenicity	Very low	Very low	Very low	Very low	Very low
Lipinski violation?	No	No	No	Yes	Yes

## Data Availability

The NMR data used to support the findings of this study are available on supplementary data.

## Supplementary Materials

Supplementary Materials Figures S1 and S2 show the ^1^H NMR and ^13^C NMR spectra of compound **1**, respectively. Figures S3 and S4 display the ^1^H NMR and ^13^C NMR spectra of compound **2**, respectively. Similarly, Figures S5 and S6 show the ^1^H NMR and ^13^C NMR spectra of compound **3**, while Figures S7 and S8 show the ^1^H NMR and ^13^C NMR spectra of compound **4**, respectively.
