# Supplementary material for: Bioassay-Guided Isolation of Antiplasmodial Compounds from Hypericum lanceolatum Lam. (Hypericaceae) and Their Cytotoxicity and Molecular Docking
Source: Biomed Res Int. 2023 May 29;2023:4693765. doi: 10.1155/2023/4693765 (PMC10241580; doi:10.1155/2023/4693765)
Supplement: Supplementary Materials — Figures S1 and S2 show the 1H NMR and 13C NMR spectra of compound 1, respectively. Figures S3 and S4 display the 1H NMR and 13C NMR spectra of compound 2, respectively. Similarly, Figures S5 and S6 show the 1H NMR and 13C NMR spectra of compound 3, while Figures S7 and S8 show the 1H NMR and 13C NMR spectra of compound 4, respectively. [file 4693765.f1.docx]

**SUPPLEMENTARY MATERIAL**

**Bioassay-guided isolation of antiplasmodial compounds from *Hypericum lanceolatum* Lam. (Hypericaceae), their cytotoxicity and molecular docking**

Gervais Mouthé Happi^1^*^,^**, Sikiru Akinyeye Ahmed^2^, Guy Paulin Mouthé Kemayou^3^, Shina Salau^2^, Liliane Clotilde Dzouemo^4^, Klev Gaïtan Sikam^4^, Mireille Towa Yimtchui^4^, Jean Duplex Wansi^4^

*^1^ Department of Chemistry, Higher Teacher Training College, The University of Bamenda, P.O Box 39 Bambili, Cameroon*

*^2^ Department of Chemistry and Industrial Chemistry, Kwara State University, Malete, P.M.B 1530 Ilorin, 23431, Nigeria*

*^3^ Department of Organic Chemistry, Faculty of Sciences, University of Yaounde I, P.O. Box 812 Yaounde, Cameroon*

*^4^ Department of Chemistry, Faculty of Sciences, University of Douala, P.O. Box 24157 Douala, Cameroon*

---

***Correspondence :***

Gervais Mouthé Happi ([gervais20022003@yahoo.fr](mailto:gervais20022003@yahoo.fr))

**Contents**

1. **Physical aspects and spectroscopic data of isolated compounds**

1,6-Dihydroxyxanthone (**1**)

Norathyriol (**2**)

Betulinic acid (**3**)

Ursolic acid (**4**)

1. **Spectra of the isolated compounds**

**Figure S1** : ^1^H NMR spectrum of compound **1** in MeOD (500 MHz)

**Figure S2** : ^13^C NMR Spectrum of compound **1** in MeOD (500 MHz)

**Figure S3** : ^1^H NMR spectrum of compound **2** in MeOD (500 MHz)

**Figure S4** : HMBC Spectrum of compound **2** in MeOD

**Figure S5** : ^1^H NMR Spectrum of compound **3** in CDCl_3_+MeOD (1:1) (500 MHz)

**Figure S6** : ^13^C NMR Spectrum of compound **3** in CDCl_3_+MeOD (1:1) (125 MHz)

**Figure S7** : ^1^H NMR Spectrum of compound **4** in Pyridine-*d*5 (500 MHz)

**Figure S8** : ^13^C NMR Spectrum of compound **4** in Pyridine-*d*5 (125 MHz)

1. ***Physical aspects and spectroscopic data of isolated compounds***

1,6-Dihydroxyxanthone (**1**) : Orange solid ‒ ^1^H NMR (500 MHz, CDCl_3_) *δ*: 12.8 (1H, s, OH-1), 7.65 (1H, t, *J* = 8.5 Hz, H-3), 7.55 (1H, d, *J* = 1.8 Hz, H-5), 7.48 (1H, d, *J* = 8.6 Hz, H-8), 7.35 (1H, dd, *J* = 8.6, 1.8 Hz, H-7), 6.98 (1H, dd, *J* = 8.6, 2.2 Hz, H-4), 6.76 (1H, dd, *J* = 8.4, 2.2 Hz, H-2). ^13^C NMR (125 MHz, CDCl_3_) *δ*: 182.2 (C-9), 161.5 (C-1), 156.4 (C-4a), 154.5 (C-10a), 150.0 (C-6), 136.4 (C-3), 124.9 (C-7), 120.7 (C-8a), 118.8 (C-8), 109.2 (C-2), 108.0 (C-9a), 107.8 (C-5), 106.6 (C-4).

Norathyriol (**2**) : Brown powder ‒ ^1^H NMR (500 MHz, CD_3_OD) *δ*: 7.46 (1H, s, H-8), 6.85 (1H, s, H-5), 6.32 (1H, d, *J* = 2.2 Hz, H-2), 6.17 (1H, d, *J* = 2.2 Hz, H-4). ^13^C NMR (125 MHz, CD_3_OD) *δ*: 179.7 (C-9), 164.9 (C-3), 162.9 (C-4a), 158.0 (C-1), 153.9 (C-6), 151.6 (C-10a), 143.3 (C-7), 112.2 (C-8a), 107.6 (C-8), 102.9 (C-9a), 101.8 (C-5), 97.2 (C-4), 93.3 (C-2).

Betulinic acid (**3**) : White powder ‒ ^1^H NMR (CD_3_OD+CDCl_3_, 500 MHz) *δ*: 4.69 (1H, m, H-29a), 4.57 (1H, m, H-29b), 3.33 (1H, ddd, *J* = 5.0, 16.9, 10.5 Hz, H-19), 3.13 (1H, dd, *J* = 11.5, 4.7 Hz, H-3), 3.01 (1H, m, H-16b), 2.29 (1H, m, H-13), 2.26 (1H, m, H-15b), 2.23 (1H, m, H-1b), 1.95 (1H, m, H-12b), 1.89 (1H, m, H-18), 1.68 (3H, s, H-30), 1.57 (2H, m, H-2), 1.43 (1H, m, H-6a), 1.42 (1H, m, H-6b), 1.40 (1H, m, H-16a), 1.35 (1H, m, H-11a), 1.32 (2H, m, H-7), 1.31 (1H, m, H-15a), 1.29 (1H, m, H-9), 1.25 (1H, m, H-11b), 1.04 (1H, m, H-12a), 0.98 (3H, s, H-26), 0.95 (6H, s, H-26, H-27), 0.89 (1H, m, H-1a), 0.83 (3H, s, H-23), 0.74 (3H, s, H-25), 0.68 (1H, m, H-5); ^13^C NMR (CD_3_OD+CDCl_3_, 125 MHz) *δ*: 178.9 (C-28), 150.6 (C-20), 109.2 (C-29), 78.5 (C-3), 56.2 (C-17), 55.4 (C-5), 50.6 (C-9), 49.1 (C-19), 46.9 (C-18), 42.3 (C-14), 40.6 (C-8), 39.7 (C-4), 38.7 (C-13), 38.3 (C-1), 37.0 (C-10), 36.9 (C-22), 34.2 (C-7), 32.1 (C-16), 30.4 (C-15), 29.5 (C-21), 27.6 (C-23), 26.7 (C-2), 25.5 (C-12), 20.8 (C-11), 18.8 (C-30), 18.2 (C-6), 15.8 (C-25), 15.6 (C-24), 15.1 (C-27), 14.3 (C-26).

Ursolic acid (**4**) : White amorphous powder ‒ ^1^H NMR (C_5_D_5_N, 500 MHz) *δ*: 5.49 (1H, t, *J* = 3.8 Hz, H-12), 3.45 (1H, dd, *J* = 10.2, 3.2 Hz, H-3), 2.62 (1H, d, *J* = 6.2 Hz, H-18), 1.32 (1H, m, H-19), 1.36 (1H, m, H-20), 1.24 (3H, s, H-27), 1.22 (3H, s, H-24), 1.05 (3H, s, H-25), 1.02 (3H, s, H-26), 1.00 (3H, d, *J* = 6.3 Hz, H-30), 0.94 (3H, d, *J* = 6.4 Hz, H-29), 0.89 (3H, s, H-23), 0.85 (1H, dd, *J* = 9.8, 4.4 Hz, H-5). ^13^C NMR (C_5_D_5_N, 125 MHz) *δ*: 179.7 (C-28), 139.0 (C-13), 125.4 (C-12), 77.9 (C-3), 55.6 (C-5), 53.3 (C-18), 47.8 (C-9), 42.3 (C-17), 39.7 (C-4), 39.3 (C-14), 39.2 (C-8), 39.1 (C-20), 38.8 (C-1), 37.2 (C-19), 37.0 (C-10), 33.4 (C-7), 30.8 (C-24), 28.6 (C-15), 28.4 (C-2), 27.9 (C-27), 24.7 (C-22), 23.7 (C-21), 23.4 (C-11), 23.4 (C-16), 21.2 (C-30), 18.6 (C-6), 17.3 (C-29), 17.2 (C-26), 16.4 (C-23), 15.4 (C-15).

1. ***Spectra of the isolated compounds***

**Figure S1** : ^1^H NMR spectrum of compound **1** in MeOD (500 MHz)

**Figure S2** : ^13^C NMR Spectrum of compound **1** in MeOD (500 MHz)

**Figure S3** : ^1^H NMR spectrum of compound **2** in MeOD (500 MHz)

**Figure S4** : HMBC Spectrum of compound **2** in MeOD

**Figure S5** : ^1^H NMR Spectrum of compound **3** in CDCl_3_+MeOD (1:1) (500 MHz)

**Figure S6** : ^13^C NMR Spectrum of compound **3** in CDCl_3_+MeOD (1:1) (125 MHz)

**Figure S7** : ^1^H NMR Spectrum of compound **4** in Pyridine-*d*5 (500 MHz)

**Figure S8** : ^13^C NMR Spectrum of compound **4** in Pyridine-*d*5 (125 MHz)
